# Functional Translocation of Broca's Area in a Low-Grade Left Frontal Glioma: Graph Theory Reveals the Novel, Adaptive Network Connectivity

**DOI:** 10.3389/fneur.2019.00702

**Published:** 2019-07-02

**Authors:** Qiongge Li, Jian W. Dong, Gino Del Ferraro, Nicole Petrovich Brennan, Kyung K. Peck, Viviane Tabar, Hernán A. Makse, Andrei I. Holodny

**Affiliations:** ^1^Levich Institute and Physics Department, City College of New York, New York, NY, United States; ^2^Department of Radiology, Memorial Sloan Kettering Cancer Center, New York, NY, United States; ^3^Brain Tumor Center, Memorial Sloan Kettering Cancer Center, New York, NY, United States; ^4^Brigham and Women's Hospital, Harvard Medical School, Boston, MA, United States; ^5^Department of Medical Physics, Memorial Sloan Kettering Cancer Center, New York, NY, United States; ^6^Department of Neurosurgery, Memorial Sloan Kettering Cancer Center, New York, NY, United States; ^7^Department of Radiology, Weill Medical College of Cornell University, New York, NY, United States; ^8^Neuroscience, Weill Medical College of Cornell University, New York, NY, United States

**Keywords:** graph theory, glioma, fMRI, language function reorganization, brain tumor surgery

## Abstract

We describe frontal language reorganization in a 50–60 year-old right-handed patient with a low-grade left frontotemporal insular glioma. Pre-operative fMRI revealed robust activation in the left superior temporal gyrus (Wernicke Area, WA) and in the right inferior frontal gyrus (right anatomical homolog of Broca Area, BA). Intra-operative cortical stimulation of the left inferior frontal gyrus and adjacent cortices elicited no speech deficits, and gross total resection including the expected location of BA resulted in no speech impairment. We employed statistical inference methods to reconstruct the functional brain network and determined how different brain areas connect with one another. We found that the right homolog of the BA in this patient functionally connected to the same areas as the left BA in a typical healthy control. As opposed to the functional connection of the left BA in a healthy brain, the right BA did not connect directly with the left WA, but connected indirectly, mediated by the pre-Supplementary Motor Area and the Middle Frontal Gyrus. This case illustrates that pre-surgical fMRI may be used to identify atypical hemispheric language reorganization in the presence of brain tumor and that network theory opens the possibility for future insight into the neural mechanism underlying the language reorganization.

## Background

The recovery of central nervous system function following an insult is poorly understood (1). How brain function reorganizes and how various brain structures involved in reorganization interconnect and interact with one another following an adverse event remain a matter of speculation (2–8).

We present a case of a left frontal glioma in a 100% right-handed patient without language deficits in whom the pre-operative fMRI showed an apparent reorganization of the left frontal language area (Broca's Area—BA) to the right. The fMRI findings were substantiated by direct cortical stimulation and neurosurgical resection of the expected location of left frontal language area.

The organization of language function in the human brain is currently seen more as a network of interconnecting nodes (5, 6, 9–12). To better understand how expressive language function in the patient reorganized—including which parts of the brain assumed the function of the left BA and how they were incorporated with the other language areas—we applied a mathematical approach—graph theory (11–18).

## Case Presentation

### History and Presentation

A 50–60 (exact age anonymized for patient confidentiality) year old patient (gender anonymized for patient confidentiality) presented for neurosurgery consultation of a recently diagnosed left frontotemporal insular lesion. A prior biopsy performed at an outside institution revealed a low-grade mixed oligoastrocytoma. The patient denied speech difficulty. An MRI showed a non-enhancing lesion measuring 9.1 × 4.9 cm within the left frontal and temporal lobes, including the insula and extending into the left basal ganglia. The patient was 100% right handed by the Edinburg Handedness Inventory (19) and scored 57/60 (normal range: 49–59) in the Boston Naming Test (20).

### First Pre-surgical fMRI Scan, and First Surgery

fMRI was obtained to localize and lateralize speech. Two fMRI language tasks were administered—verbal fluency and semantic fluency. Scanning was performed on 1.5T scanner (GE Healthcare, Milwaukee, Wisconsin) using an 8-channel head coil. Functional MRI data were acquired with a single shot gradient echo echo-planar imaging (EPI) sequence (TR/TE = 4,000/40 ms; 128 × 128 matrix; 4.5 mm thickness, 32 slices covering whole brain). Functional data matching T1-weighted (TR = 600 ms; TE = 8 ms; thickness = 4.5 mm) and T2-weighted (TR = 4,000 ms; TE = 102 ms; thickness = 4.5 mm) spin-echo axial slices, covering the whole brain, were obtained. 3D T1-weighted images with a spoiled gradient-recalled-echo sequence (TR = 22 ms, TE = 4 ms, 256 × 256 matrix, 30° flip angle, 1.5 mm thickness) were also acquired. There were 90 images per task consisting of 5 images of activation (20 s) followed by 10 images of rest (40 s) repeated 6 times (6 min total). Image processing and analysis were performed using Analysis of Functional Neuroimaging (AFNI) (21). Details of the fMRI technique are described in detail here (22).

**Figure 1:**
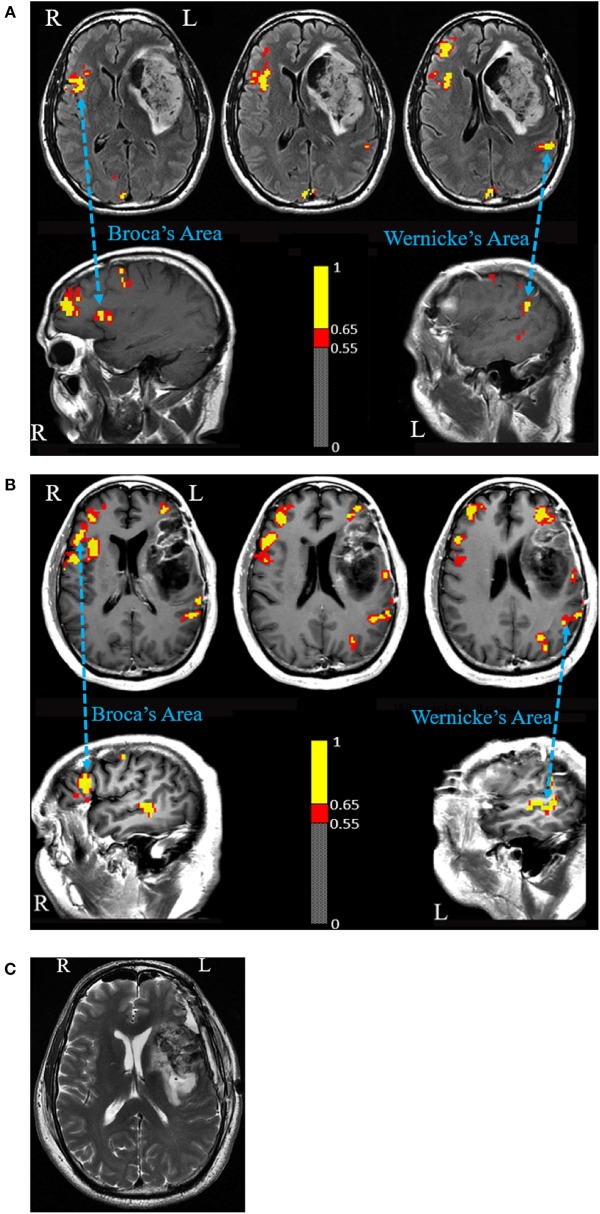
**(A)** Language fMRI prior to first surgery. A semantic fluency task fMRI shows activated Broca's Area homolog in the right hemisphere and Wernicke's Area in the left hemisphere. The figure shows voxels activated at a correlation coefficient of 0.54 (*p* = 3.8 × 10^−8^) or higher. **(B)** Language fMRI prior to second surgery. A verbal fluency task shows activated Broca's Area homolog in the right hemisphere and Wernicke's Area in the left hemisphere. Activation was also seen anterior and posterior to the expected Broca's Area in the left hemisphere. Intraoperative cortical stimulation elicited no speech disturbance in the anterior margin and motor-related disturbances in the posterior margin. The figure shows voxels activated at a correlation coefficient of 0.55 (*p* = 1.7 × 10^−7^) or higher. **(C)** Post-operative MRI scan shows a gross total resection including the expected anatomical region of Broca's Area.

In the semantic fluency task, activation was observed in the anatomical Wernicke's Area (WA) in the left hemisphere and in Broca's Area (BA) homolog in the right hemisphere (Figure 1A). No fMRI activation was identified in the expected location of the BA in the left hemisphere.

The patient underwent an MRI-assisted awake craniotomy for language mapping and tumor resection. Direct cortical stimulation (DCS) was performed with the Ojemann bipolar stimulator (Radionics Inc., Burlington, MA), providing 1 s trains of 0.5 ms square wave pulses at 60 Hz. Dysarthria and facial fasciculations were noted upon stimulation of the inferior motor strip at 4 mA. Beginning at 4 mA and escalating to 8 mA, stimulation of a large region of the frontal operculum did not elicit speech arrest, paraphasia, or word finding difficulty while the patient counted numbers, recited the days of the week, months of the year, and performed an auditory responsive naming task (e.g., what do you shave with?). Multiple corticectomies through the inferior and middle frontal gyri and superior temporal gyrus were performed using the operating microscope. The rostral aspect of the middle frontal gyrus as well-areas within the anterior inferior frontal gyrus were resected up to but not including BA. Not with standing the inability to elicit speech disturbance, the posterior half of the tumor underlying BA was not resected for fear of compromising speech function. Post-operatively, the patient recovered well without speech difficulty except for one seizure with speech disturbance.

### Second Pre-surgical fMRI Scan and Second Surgery

In the absence of post-operative aphasia, a second operation was scheduled 3 months later to resect the residual tumor. A second pre-operative fMRI was performed, using the same fMRI language tasks in addition to a verb generation task. fMRI showed strong activation of the anatomical homolog of BA in the right hemisphere in addition to activation of WA in the left hemisphere (Figure 1B). The expected location of BA in the left hemisphere did not activate in any of the tasks. Two regions of peri-lesional activation however were observed anterior and posterior to the FLAIR hyper intensity and enhancement in all three tasks. The posterior activation was felt to represent tongue motor. The anterior activation was presented as a possible candidate for BA in the left hemisphere despite its anterior location.

During the second resection, language mapping via DCS of the exposed frontal lobe including the left expected BA induced no speech arrest as the patient performed tasks identical to those in the first surgery. DCS of the suspected BA candidate activated during fMRI in the anterior margin of the tumor did not elicit speech disturbance. Dysarthria and motor speech disturbances were noted upon stimulation of the inferior aspect of the motor strip.

Resection of the residual tumor began at the temporal aspect of the cavity. The resection under the operating microscope proceeded with tumor removal medially, posteriorly, and, superiorly toward the insula. Tumor resection then proceeded through the inferior frontal cortex. The patient was tested for speech continuously. Residual tumor was removed, allowing communication of the inferior and temporal aspects of the insula. Assessment of the resection by intraoperative MRI was performed, which demonstrated a gross-total resection (Figure 1C). Post-operatively, the patient did not experience speech impairment.

### Network Analysis of the fMRI Results

The above results imply a reorganization of expressive speech function. However, important questions remain, which cannot be addressed using standard fMRI analysis alone. For example, what is the new functional connectivity between the right BA homolog and the left WA, as there is no known direct anatomical connection between them? What is the functional connectivity between the right BA and the additional secondary language areas? To elucidate these questions, we employed a well-established statistical inference method (11, 12, 15, 17, 18, 23) to construct a functional brain network starting from the fMRI activity, previously used in a collaborative work based on memory brain networks in rodents.

To construct the functional links connecting fMRI activated voxels in a network architecture we proceeded in two steps (13, 14, 23). First, we computed the functional connectivity matrix C and inferred a sparse representation of this matrix, indicate as J. The non-zero entries of the sparse matrix J are the *effective* functional links connecting the active voxels within a network. Second, we grouped the voxels in clusters based on their anatomical location. Details of this approach are described in literature (14).

**Figure 2:**
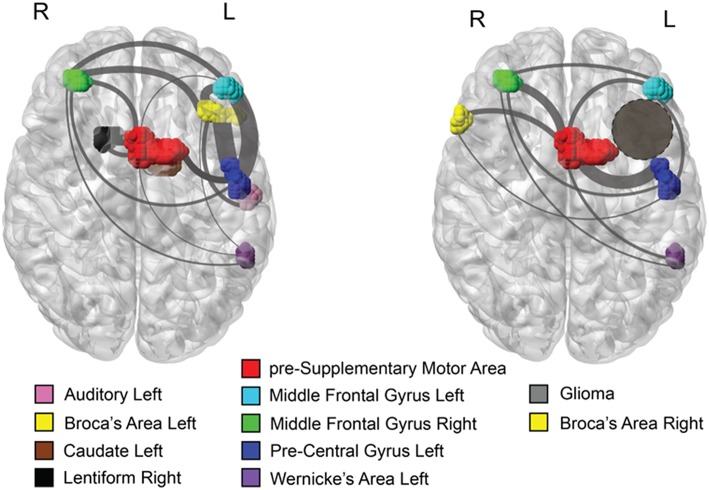
Axial views of the brain with active fMRI areas and links obtained by statistical inference. (**Left**) Brain network topology inferred from a semantic fMRI task for a typical healthy control subject. The primary language areas, BA and WA, are identified in the left hemisphere and are functionally connected. (**Right**) the network consistently found across all the fMRI scans for the patient with left insular glioma. The homolog of the BA on the right hemisphere becomes statistically active and connects with several other active brain areas, implicating a functional reorganization of the brain areas involved in a language task. As opposed to the typical healthy brain, this patient's right BA does not connect directly with the left WA. Functional communication between these two areas is conveyed through a common area to which they are both linked, the pre-SMA. Gray lines represent functional connectivity, line thickness is proportional to the strength of connectivity, whereas their arrangement is made to facilitate visualization. In both panels, colors indicate different brain regions as reported in the legend.

For comparison, we constructed a brain network for a typical healthy subject, who performed the same fMRI tasks as the patient (Figure 2, left panel), where the active brain areas in the fMRI scan were placed on their anatomical location, and the links represented functional connectivity. In the normal example, the left BA is functionally connected with the left WA, as expected from the structural fibers connecting these two areas (arcuate fasciculus). Furthermore, the left BA forms functional connections with other brain areas including the left pre-SMA, Pre-Central Gyrus (PCG), and Middle Frontal Gyrus (MFG). Figure 2, right panel, shows the functional network for the patient with the left insular glioma. The left BA is infiltrated by the tumor and consequently its fMRI activation is suppressed. We found that its anatomical homolog on the right hemisphere activates and functionally connects with the left PCG, the pre-SMA, and the right MFG. In healthy controls, these structures are connected to left BA rather than the right. As opposed to typical healthy controls, this patient's right BA does not connect directly with the left WA. The channel of information between these two important areas for language production passes indirectly through the pre-Supplementary Motor Area (pre-SMA) and the Middle Frontal Gyrus (MFG).

## Discussion

We presented a case of expressive language reorganization with fMRI and DCS results that were consistent with clinical observation. Previous studies have shown that approximately 96% of right-handed people have language function in the left hemisphere (24, 25). In our right-handed patient, fMRI indicated an expected WA in the left hemisphere, but the expected BA in the left hemisphere, infiltrated by tumor, did not activated. DCS of the left expected BA did not elicit speech impairment. Gross-total resection of the tumor in left anatomical BA region also did not result in speech impairment. Taken together, results from fMRI, DCS, surgery, and clinical observation showed that the patient experienced a reorganization of expressive speech function to regions outside the left BA region, with fMRI suggesting that the right anatomical BA homolog as the candidate for reorganized speech function. Functional network analysis illustrates that the right BA homolog functionally connects with the same brain areas to which the left BA is usually functionally linked, except for the left WA. Instead, the right BA does not connect directly with the left WA, but other apparent indirect connections appear, including through the pre-SMA and the right MFG.

Previous literature suggested that slow growing lesions can trigger compensatory mechanisms that resulted in a shift of expressive language function (e.g., left BA) to the right hemispheric homolog. For example, cases of translocation of BA have previously been reported in a patient with a low-grade tumor (26) and in pediatric epilepsy patients (27). Cases of receptive language function reorganization to the contralateral hemisphere have also been reported (7). Another study found that masses occupying BA resulted in a significantly lower language lateralization index compared to healthy controls, with a shift of fMRI language activation toward the non-dominant right hemisphere (28). In patients with masses in primary language areas, these studies demonstrated various degrees of language shift to the contralateral hemisphere. While the exact neural mechanisms of reorganization is unclear, other studies have also found that slow growing primary brain tumors afford more time for functional reorganization to occur than do acute lesions, thereby allowing eloquent cortices to redistribute around tumors or to the contralateral hemisphere (29). Shaw suggested that patients with left frontal tumors with fMRI evidence of cortical reorganization, performed significantly better on the Boston Naming Test, a clinical measure of aphasia (30). These findings support the idea of effective plasticity of higher cortical functions such as language. Without effective functional reorganization in the present patient, a resection of BA would have resulted in severe speech deficits.

There are several limitations to this case report. One prior meta-analysis of relevant literature (31) found that studies are not unequivocal in their conclusion regarding the reliability of fMRI in mapping language areas prior to surgery, with sensitivity ranging from 59 to 100% and a specificity ranging from 0 to 97%. Namely, the meta-analysis has found that prior studies utilized different language tasks, different magnet strength, analysis software, and analysis paradigms, as well as different intraoperative techniques to validate the concordance of the two brain mapping methods (fMRI and DCS). Additionally, different lesions (high grade and local grade gliomas, vascular lesions, epileptic lesions) are potential confounders.

Despite the limitations, this case report illustrates that fMRI can identify atypical hemispheric dominance in pre-surgical planning in brain tumor patients and that the application of graph theory can help elucidate the new functional connections formed by the various language centers. Additional further work such as with resting state fMRI could be considered for further validation, as recent studies demonstrate strong concordance with DCS and task based fMRI (32).

## Data Availability

The datasets generated for this study are available on request to the corresponding author. The data is available at: https://www-levich.engr.ccny.cuny.edu/webpage/hmakse/brain/.

## Ethics Statement

Our institution, MSKCC, has a general consent signed by every patient that allows their clinical material, after appropriate anonymization, to be used for purposes of research and education. MSKCC does not have a mechanism for obtaining a dedicated informed consent for case reports (as opposed to prospective and retrospective studies) as it is assumed that such cases are covered under the above policy.

## Author Contributions

JD: manuscript drafting, data interpretation, figure generations, and manuscript submission. QL: manuscript drafting, data interpretation, algorithms development, and figure generations. GDF: data interpretation, manuscript drafting, and algorithms development. NP and VT: data acquisition. KP: data acquisition and interpretation. HM and AH: conception and study design and guarantor of integrity. This current case report involves the integration of expertise in multiple advanced fields, all of which were necessary for the completion of the present manuscript. These include: physics/mathematics (QL, GDF, and HM), neuroradiology (JD and AH), medical physics (KP), and neurosurgery (VT). QL and GDF: software development.

### Conflict of Interest Statement

The authors declare that the research was conducted in the absence of any commercial or financial relationships that could be construed as a potential conflict of interest.
